# A phase Ib trial of docetaxel, carboplatin and erlotinib in ovarian, fallopian tube and primary peritoneal cancers

**DOI:** 10.1038/sj.bjc.6604371

**Published:** 2008-05-27

**Authors:** P A Vasey, M Gore, R Wilson, G Rustin, H Gabra, J-P Guastalla, E P Lauraine, J Paul, K Carty, S Kaye

**Affiliations:** 1 CR-UK Clinical Trials Unit, Beatson Oncology Centre, Western Infirmary, Dumbarton Road, Glasgow, Scotland G11 6NT, UK; 2 Royal Marsden Hospital, Fulham Road, London SW15 3SW, UK; 3 Belfast City Hospital, Lisburn Road, Belfast, Ireland BT9 7AB, UK; 4 Mount Vernon Hospital, Rickmansworth Road, Northwood, Hertfordshire HA6 2RN, UK; 5 Western General Hospital, Crewe Road South, Edinburgh, Scotland EH4 2XU, UK; 6 Centre Léon-Bérard, rue Laennec, 69373, Lyon, Cedex 08, France; 7 Hopital de L’Hotel-Dieu, place du Parvis Notre-Dame, 75181 Paris Cedex 4, France; 8 Royal Marsden Hospital, Downs Road, Sutton, Surrey SM2 5PT, UK; 9 Division of Medicine, University of Queensland, Brisbane Q4029, Australia

**Keywords:** docetaxel, carboplatin, erlotinib, HER1/EGFR, gynaecological, cancer

## Abstract

The safety and maximum tolerated dose (MTD) of erlotinib with docetaxel/carboplatin were assessed in patients with ovarian cancer. Chemonaive patients received intravenous docetaxel (75 mg m^−2^) and carboplatin (area under the curve 5) on day 1 of a 3-week cycle, and oral erlotinib at 50 (cohort 1), 100 (cohort 2a) or 75 mg day^−1^ (cohort 2b) for up to six cycles. Dose-limiting toxicities were determined in cycle 1. Forty-five patients (median age 59 years) received treatment. Dose-limiting toxicities occurred in 1/5/5 patients (cohorts 1/2a/2b). The MTD of erlotinib in this regimen was determined to be 75 mg day^−1^ (cohort 2b; the erlotinib dose was escalated to 100 mg day^−1^ in 11 out of 19 patients from cycle 2 onwards). Neutropaenia was the predominant grade 3/4 haematological toxicity (85/100/95% respectively). Common non-haematological toxicities were diarrhoea, fatigue, nausea and rash. There were five complete and seven partial responses in 23 evaluable patients (52% response rate). Docetaxel/carboplatin had no measurable effect on erlotinib pharmacokinetics. In subsequent single-agent maintenance, erlotinib was given at 100–150 mg day^−1^, with manageable toxicity, until tumour progression. Further investigation of erlotinib in epithelial ovarian carcinoma may be warranted, particularly as maintenance therapy.

In 2002, the estimated worldwide incidence of ovarian cancer was almost 205 000 with nearly 125 000 women dying from this disease (Globocan, 2002). Current treatment for advanced ovarian cancer is cytoreductive surgery followed by six cycles of platinum-based chemotherapy. In phase III trials, paclitaxel/carboplatin is as effective as paclitaxel/cisplatin but is less toxic and also has quality of life (QoL) benefits (Neijt *et al*, 2000; Ozols *et al*, 2003; du Bois *et al*, 2003). However, paclitaxel/carboplatin can produce significant haematologic and neurologic toxicity. Thus, new therapy options are needed to improve both clinical outcome and treatment tolerance.

Docetaxel has pharmacologic and pharmacokinetic (PK) advantages over paclitaxel. In phase II trials, docetaxel had significant activity in platinum-resistant ovarian cancer (Kaye *et al*, 1997) and in paclitaxel-resistant Mullerian cancers (Vershraegen *et al*, 2000). A phase III trial demonstrated that docetaxel/carboplatin had similar efficacy to paclitaxel/carboplatin in advanced ovarian cancer; response rates: 58.7 *vs* 59.5%; progression-free survival (PFS): 15.0 *vs* 14.8 months (Vasey *et al*, 2004). Docetaxel/carboplatin induced less neurotoxicity (e.g. grade ⩾2 neurosensory toxicity: 11 *vs* 30%, *P*<0.001) and more haematologic toxicity (e.g. grade 3/4 neutropaenia: 94 *vs* 84%, *P*<0.001) but with improved QoL parameters compared with paclitaxel/carboplatin. Thus, docetaxel/carboplatin could represent an alternative first-line chemotherapy regimen for patients with advanced ovarian cancer (du Bois *et al*, 2005), although not currently registered for this indication.

Improved understanding of tumorigenesis has resulted in novel antitumour agents acting on specific cellular targets being developed. One therapeutic target is the human epidermal growth factor receptor (EGFR) (Arteaga 2003). Increased EGFR expression occurs in approximately 70% of ovarian tumours (Kohler *et al*, 1992). In some cancers, particularly ovarian, dysregulation of EGFR is associated with poor prognosis (Nicholson *et al*, 2001).

Erlotinib (Tarceva^®^) is a highly potent, orally active inhibitor of the tyrosine kinase (TK) region of EGFR. The dose of erlotinib recommended for further study (150 mg day^−1^) was identified in a key phase I PK study (Hidalgo *et al*, 2001). Initial phase II clinical trials demonstrated varied antitumour activity of erlotinib monotherapy in a wide range of tumours, for example, head and neck (Soulières *et al*, 2004), lung (Perez-Soler *et al*, 2004) and colorectal cancer (Townsley *et al*, 2006). More recently, the importance of tumour characteristics in predicting response to erlotinib has been recognised (notably, mutations in the EGFR TKI domain) (Uramoto and Mitsudomi, 2007). Erlotinib monotherapy significantly prolonged survival of patients with chemorefractory advanced NSCLC compared with best supportive care in a large randomised trial (Shepherd *et al*, 2005).

In patients with refractory, recurrent, EGFR-positive epithelial ovarian tumours who had failed prior taxane and/or platinum-based chemotherapy, erlotinib monotherapy was generally well tolerated (Gordon *et al*, 2005). In this phase II trial, the objective response rate was 6% and 15 patients (44%) had stable disease. Although this is not suggestive of significant efficacy as monotherapy in ovarian carcinoma, the addition of erlotinib to chemotherapy has the potential to improve outcomes. Combining agents with different modes of action and a limited overlap of toxicity profiles should improve therapeutic strategies for patients with advanced cancer. Preclinically, erlotinib with chemotherapy showed additive or synergistic antitumour effects in human xenograft models (e.g. Higgins *et al*, 2004; Ouchi *et al*, 2006).

The primary objectives of the present study were to determine the safety, tolerability and maximum tolerated dose (MTD) of daily oral erlotinib in combination with 3-week cycles of docetaxel/carboplatin as first-line treatment for patients with epithelial ovarian cancer, fallopian tube cancer or primary peritoneal cancer. Other objectives included a PK analysis of erlotinib and the cytotoxics, plus documentation of antitumour activity. Cytotoxic doses were docetaxel (Taxotere^®^, 75 mg m^−2^) and carboplatin (Paraplatin^®^, AUC 5), as this combination has proven efficacy in patients with ovarian cancer (Vasey *et al*, 2004). Preliminary data in heavily pretreated patients indicated increased myelogenous toxicity with erlotinib (>100 mg day^−1^) when used in combination with docetaxel 75 mg m^−2^ (Forouzesh *et al*, 2002), so for this trial, a lower initial dose of erlotinib (50 mg day^−1^) was selected.

## PATIENTS AND METHODS

### Patient population

Eligible patients were women aged ⩾18 years of age with histologically confirmed epithelial ovarian, fallopian tube or primary peritoneal carcinoma. Additional inclusion criteria were as follows: International Federation of Gynecologic Oncology (FIGO) stage III–IV disease; Eastern Cooperative Oncology Group (ECOG) performance status (PS) 0–2; no prior exposure to chemo- or radiotherapy; ⩽8 weeks following surgery (debulking surgery was not an entry requirement; however, patients not considered operable must have had appropriate pathology on biopsy). Key exclusion criteria included symptomatic peripheral neuropathy; inadequate renal, hepatic, cardiopulmonary or haematologic function; severe and/or uncontrolled comorbidity; and prior sensitivity to docetaxel.

The study was approved by multicentre and local research ethics committees, and was conducted according to the recommendations of the Declaration of Helsinki. All patients gave written informed consent.

### Trial objectives, design and drug treatment

The primary objective was to determine the safety, tolerability and MTD of daily oral erlotinib in combination with docetaxel and carboplatin in patients with advanced ovarian cancer. The secondary objectives included to evaluate the PKs of erlotinib, docetaxel and carboplatin when administered in combination and to conduct a preliminary investigation of the antitumour activity of this combination regimen.

This trial was a phase Ib, open-label, dose-escalation study. Patients (12 planned/cohort) were enrolled sequentially into a cohort. Toxicities during the first treatment cycle were used to determine the tolerability of the dosage regimen for that cohort; data were evaluated when all 12 patients had completed cycle 1. Escalation of the erlotinib dose for the next cohort only took place if less than 4 of 12 patients experienced a dose-limiting toxicity (DLT). If four or more patients had DLTs, then patients were recruited into the relevant interim cohort. The initial dose of erlotinib was 50 mg day^−1^.

In cohort 1, patients were randomised to receive erlotinib in either cycle 1 or 2, but not in cycles 2 and 1, respectively. This was carried out to assess the effect of erlotinib on nadir neutrophil counts in a crossover design. Thereafter, patients received erlotininb in cycles 3–6 as usual.

Erlotinib hydrochloride (25, 100 and 150 mg tablets) was supplied by F Hoffmann-La Roche Ltd (Basel, Switzerland). Commercially available preparations of docetaxel and carboplatin were supplied. On day 1 of each cycle, docetaxel (Taxotere, 75 mg m^−2^, Sanofi-Aventis, Bridgewater, NJ, USA) was administered as a 1-h intravenous infusion in 250 ml of 0.9% saline, immediately followed by carboplatin (Paraplatin, AUC 5, Bristol-Myers Squibb, New York, NY, USA) as a 1-h intravenous infusion in 500 ml of 5% glucose. The dose of carboplatin was determined using the Calvert formula and, in most cases, glomerular filtration rate (GFR) was obtained using radioisotope (51Cr-labelled ethylenediaminetetraacetic acid (51CrEDTA)) measurement. Pre-medication with dexamethasone (8 mg twice daily for 3 days starting the day before docetaxel) was given, as well as prophylactic anti-emetics with the chemotherapy. Erlotinib was taken orally, beginning 7 days before the first dose of chemotherapy. On days of concomitant administration, erlotinib was taken at least 1 h before chemotherapy (except when PK samples were being collected).

Treatment delays and dose reductions of docetaxel and carboplatin were permitted based on predefined criteria. Occurrences of severe rash or diarrhoea not sufficiently controlled by supportive treatment resulted in dose reduction of erlotinib by 25 or 50 mg day^−1^, followed by dose interruption if necessary.

The planned duration of treatment was six 3-week cycles of chemotherapy. Erlotinib monotherapy was permitted after chemotherapy until disease progression or unacceptable toxicity. During this period, the dose of erlotinib was increased from 50 to 150 mg day^−1^ by 25 mg week^−1^ increments.

### Determination of the MTD

The MTD was defined as the dose below which, during the first cycle with erlotinib, DLTs were caused in >1/3 of patients. A DLT was any of the following: grade 4 neutropaenia for >7 days; febrile neutropaenia (absolute neutrophil count <1 × 10^9^ l^−1^; temperature ⩾38.5°C); grade 4 thrombocytopaenia (<10 × 10^9^ l^−1^) associated with bleeding or requiring platelet transfusion; grade 2 diarrhoea lasting >48 h despite loperamide treatment; any non-haematologic toxicity ⩾grade 3 (except tolerated rash and grade 3 self-limiting or medically controllable toxicity); treatment delays exceeding 1 (erlotinib) or 2 weeks (chemotherapy), as a result of lack of recovery from grade 2 toxicity.

### Safety and tolerability

At baseline, patients underwent a physical examination, chest X-ray, 12-lead electrocardiogram (ECG), GFR measurement (using 51CrEDTA or 24-h creatinine clearance), and standard blood tests (full blood count and biochemical profile). Full blood count was checked weekly. A physical examination, full blood count and biochemistry were conducted before each cycle. ECOG PS was evaluated at baseline and before each cycle.

Safety was assessed by the incidence and severity of adverse events (AEs), using the National Cancer Institute Common Toxicity Criteria (NCI-CTC) version 2.0 and changes in laboratory values. Patients were assessed for AEs prior to each cycle, and were encouraged to report any findings that occurred between hospital visits. Pre-existing conditions that worsened during the course of the study were also reported as AEs.

### Pharmacokinetics

Pharmacokinetic analysis was carried out by MDS Pharma Services (2350 Cohen Street, St Laurent, Quebec, Canada), using HPLC combined with triple mass spectrometric detection. Pharmacokinetic parameters for erlotinib were evaluated in the first cycle with erlotinib, in patients in cohort 1 only. Blood samples were collected into lithium heparin tubes pre-dosing and at 0.5, 1, 2, 3, 4, 6, 8, 10 and 24 h post-dosing on days −1, 1 and 7. Plasma samples, prepared by centrifugation within 1 h of collection, were stored frozen at −70°C until analysis.

The following PK parameters were evaluated for erlotinib: maximum observed concentration (*C*_max_), time to peak concentration (*T*_max_) and area under the curve (AUC 0–24 h). These parameters were assessed on the day prior to chemotherapy, on the day of chemotherapy and 6 days after chemotherapy.

### Antitumour efficacy

At baseline, each patient underwent a pelvic examination and an abdomino-pelvic computed tomography (CT) scan, and blood levels of the ovarian tumour marker CA-125 were assessed. Patients with palpable lesions had a pelvic examination before each cycle. After cycles 3 and 6, abdomino-pelvic CT scans were performed on patients who had (a) disease evident at baseline and (b) a negative baseline scan with CA-125 evidence of disease progression. CA-125 blood levels were measured before each treatment cycle and during follow-up. Evaluation of tumour response was based on the Response Evaluation Criteria in Solid Tumours (RECIST) and on changes in CA-125 levels. Progression-free survival was defined as time to progression or death from any cause. Progressive disease was defined as either clinical evidence of progressive disease based on either the RECIST Response Criteria for Solid Tumours or elevated CA125 levels as defined as either (a) an increase by twice the upper limit of normal for patients where the Ca-125 normalises or is never elevelated, or (b) an increase by twice the nadir value for patients with elevated pretreatment and who do not normalise values after therapy (any elevated levels have to be confirmed by repeat testing).

### Statistical methods

In this study, a planned cohort size of 12 evaluable patients in each dose escalation cohort was used, rather than the usual six. The main reason for this was to reduce the risk of the MTD being established on the basis of docetaxel/carboplatin toxicities alone. In addition, the larger sample size provided greater power to detect any potential effect of erlotinib on nadir neutrophil counts in cohort 1.

The cohort of the dose level to be taken forward to phase III was planned to be expanded to 20 patients, to allow more experience with the combination at this dose level.

For cohort 1, nadir neutrophil data and erlotinib PK were analysed using repeated measures analysis of variance (ANOVA) techniques. A log transformation was applied to the data before analysis, in order to achieve approximate normality (this transformation was based on the experience from other studies).

## RESULTS

### Patient characteristics and treatment

Forty-eight patients were registered at seven hospitals and included 15, 13 and 20 patients in cohorts 1, 2a and 2b, respectively. Three patients did not receive erlotinib and were excluded from the study. In cohort 1, one patient had a severe allergic reaction to docetaxel and another patient had complications due to a wound infection. The third patient (cohort 3) withdrew consent. Baseline patient and disease characteristics are summarised in Table 1.

Erlotinib duration ranged from 28 to 184 (cohort 1), 11 to 177 (cohort 2a) and 7 to 222 days (cohort 2b). The median erlotinib doses were 47.5, 59.1 and 75.5 mg day^−1^ (cohorts 1, 2a and 2b, respectively). Treatment-related effects resulted in erlotinib dose reduction/delay for some patients that is intolerable cutaneous toxicity: *n*=1, 2, 4; ⩾grade 3 diarrhoea: *n*=1, 1, 4; or other reasons: *n*=0, 4, 0 (cohorts 1, 2a and 2b, respectively). Erlotinib treatment was stopped during the six cycles of combination treatment for 1/13, 5/13 and 6/19 patients in cohorts 1, 2a and 2b, respectively. The reasons for this cessation were cohort 2a: patient refusal (*n*=1), grade 3 diarrhoea (*n*=2), other treatment-related reason (*n*=1) and not treatment related (*n*=1); cohort 2b: refusal (*n*=2), diarrhoea (*n*=1), rash (*n*=2), and other treatment-related reason (*n*=1).

The majority of patients (32 of 44) received all six chemotherapy cycles. Among the rest, 10 out of 12 patients received three cycles or less. The main reasons for stopping treatment early were disease progression/death from disease. Overall, 89% of cycles were administered without any delay. Most of the delays were unrelated to drug treatment (*n*=14 cycles). Ten cycles were delayed due to drug-related issues, and four of these were due to haematologic toxicity (thrombocytopaenia, *n*=3; neutropaenia, *n*=1). Full doses of carboplatin and docetaxel were administered in 96 and 94% of cycles, respectively.

### Determination of MTD

In cohort 1 (initial erlotinib dose, 50 mg day^−1^), only one patient had a DLT (grade 3 plantar–palmar erythrodysesthesia in cycle 2, which was the first cycle involving erlotinib in this patient, as they were part of the crossover phase of the study). In cohort 2a (initial erlotinib dose, 100 mg day^−1^), 5 of 13 patients (38%) had at least one DLT in the first cycle, namely persistent diarrhoea (two patients); delayed erlotinib administration because of incomplete recovery from grade 2 toxicity (four patients); and (all in one patient) grade 3 vomiting, dehydration and rash, grade 4 oesophagitis and neutropaenic sepsis. Thus, erlotinib 100 mg day^−1^ with docetaxel and carboplatin exceeded the MTD. Patients were subsequently recruited to cohort 2b.

In cohort 2b (initial erlotinib dose, 75 mg day^−1^), DLTs were observed in 5 out of 19 (26%) patients in cycle 1. One patient had grade 3 rash, whereas another had grade 3/4 febrile neutropaenia, persistent diarrhoea, and grade 3 dysphagia, dehydration, hyperglycaemia and dizziness. Three patients had erlotinib administration delayed. Among these three, one also had several grade 3 toxicities (dehydration, dysphagia, mucositis, abdominal pain and throat pain) and another had grade 3 aesthenia and dehydration.

Escalation of the dose of erlotinib (from 75 to 100 mg day^−1^) was permitted for patients in cohort 2b following the satisfactory completion of a cycle at 75 mg. Eleven of 19 patients (58%) had a dose escalation, that is, 4, 5, 1 and 1 patients starting in cycles 2, 3, 5 and 6, respectively. Twenty-three of 27 patients had their dose of erlotinib increased to 150 mg day^−1^ after chemotherapy.

### Safety and tolerability

All patients in the trial experienced at least one AE, the majority of which were mild to moderate in severity. The most common grade 3/4 non-haematologic toxicities were diarrhoea, rash, fatigue and dehydration (Table 2). As expected with docetaxel/carboplatin, most patients experienced grade 3/4 haematologic toxicity (Table 3). Neutropaenia was recorded in 85, 100 and 95% of patients in cohorts 1, 2a and 2b, respectively.

There were no clinically significant changes in the results of physical examinations, chest X-rays or ECGs. Most changes in laboratory values were grade 1 or 2 and were not dose related. There were very few grade 3/4 biochemical abnormalities (data not shown) and these were not considered clinically important or necessarily treatment related.

The data count from the crossover study conducted in cohort 1 indicated no statistically significant impact of erlotinib on nadir neutrophil counts.

### Pharmacokinetics

**Figure 1:**
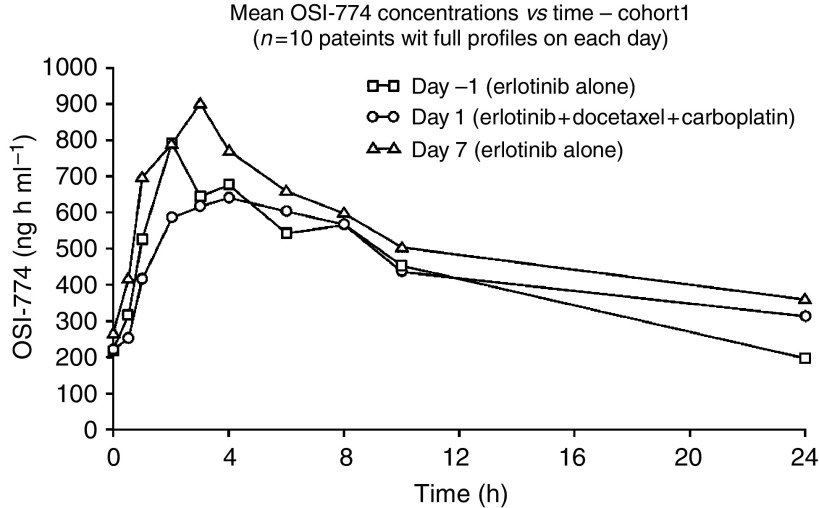
Mean plasma concentration–time curves for erlotinib alone and in combination with docetaxel and carboplatin for cohort 1.

In the first cohort, seven patients received erlotinib in cycle 1, and three in cycle 2. There were no clear differences in plasma *C*_max_, *T*_max_ or AUC_(0–24 h)_ values for erlotinib between samples taken 24 h before or 6 days after chemotherapy, or on the same day as chemotherapy (Table 4). Thus, the changes in *C*_max_, *T*_max_ and AUC_(0–24 h)_ over time (between days −1, 1 and 7) were not statistically significant (*P*=0.079, 0.410 and 0.882 respectively; repeated measures ANOVA). The mean plasma concentration–time curves for erlotinib alone and in combination with chemotherapy (Figure 1) confirm that exposure to erlotinib is unaffected by concomitant administration of docetaxel/carboplatin.

### Antitumour efficacy

Twenty-four patients had measurable disease and were evaluable for tumour response (data not shown). In total, five patients had a complete response (CR), and seven patients had a partial response (PR), giving an overall objective response rate (CR plus PR) was 12 out of 23 (52%).

### Survival

Of the patients who completed chemotherapy, 17 have since progressed and 23 have died. The median PFS for all patients was 12.5 months (95% confidence interval (CI), 9.5–15.6 months) and the median overall survival (OS) for all patients was 37.0 months (95% CI, 27.3–46.7 months). The median follow-up for living patients is 34 months (range, 25–45 months).

### Outcomes among patients who received erlotinib monotherapy after completion of chemotherapy

Of the 48 patients who started the study, 27 (56%) continued erlotinib monotherapy after completion of chemotherapy. In 23 of these patients, the dosage was escalated to 150 mg day^−1^, as planned. The median duration of treatment after chemotherapy was 8.6 months (range 2.3–32.5 months). Twelve of the 27 patients (44%) subsequently had their dose reduced or interrupted due to toxicity (skin toxicity in 10 out of 12 patients). Nevertheless, apart from alopoecia (33%), rash or desquamation (22%) and other skin complaints (11%), the incidence of severe toxicity (grade 2 or greater) was low.

Twenty-two patients (81%) stopped erlotinib because of progressive disease and another three (11%) because of skin toxicity. Two patients (7%) are continuing to receive erlotinib without evidence of progression, both at a daily dose of less than 150 mg (due to skin toxicity). The median PFS in patients receiving erlotinib monotherapy was 14.8 months (95% CI): 12.6–17.1 months) and the median OS was 37.0 months (95% CI: 31.6–42.4 months).

## DISCUSSION

This phase Ib study assessed the feasibility of combining erlotinib with docetaxel and carboplatin as first-line therapy in patients with advanced Mullerian cancers. The primary objective of this trial was to determine the MTD of erlotinib with standard doses of chemotherapy. In addition, this paper is the first report on the PK of erlotinib combined with these agents.

The MTD of erlotinib was defined as 75 mg day^−1^ when administered with docetaxel (75 mg m^−2^) and carboplatin (AUC 5) on day 1 of each 21-day cycle. The nine DLTs observed in 5 out of 13 patients in cohort 2a (100 mg day^−1^ erlotinib) included persistent diarrhoea and vomiting as well as dose cessation of erlotinib. Interestingly, most patients (11/19, 58%) in cohort 2b had their erlotinib dose escalated from 75 to 100 mg day^−1^ during cycles 2–6, indicating that the higher dose could be reasonably well tolerated when used in combination with chemotherapy, after an initial lower dose.

The toxicity profile of erlotinib/docetaxel/carboplatin combined was consistent with the known toxicities of the individual drugs. All patients had at least one AE, but most of the toxicities were mild to moderate in severity. The majority of patients had grade 3/4 haematologic toxicities in response to chemotherapy, with neutropaenia (93%) and leucopoenia (87%) being the most common findings. Severe thrombocytopoenia (13%) and anaemia (2%) occurred much less frequently. The present results, in keeping with the key toxicities, are reported for first-line docetaxel/carboplatin in patients with advanced ovarian carcinoma (Vasey *et al*, 2004), that is 94, 9 and 11% grade 3/4 neutropaenia, thrombocytopoenia and anaemia, respectively.

The other most common treatment-related grade 3/4 AEs in the current study were diarrhoea, rash, febrile neutropaenia/infection (subsequent to severe chemotherapy-induced neutropaenia), fatigue and dehydration. Diarrhoea is common with docetaxel/carboplatin (Vasey *et al*, 2004) and with erlotinib (Shepherd *et al*, 2005). Similarly, fatigue is common to both chemotherapy and erlotinib (Vasey *et al*, 2004; Shepherd *et al*, 2005). The rash observed in the present trial resulted from the erlotinib therapy, and has been reported in trials with erlotinib alone or in combination with chemotherapy in various tumour types (Gordon *et al*, 2005; Moore *et al*, 2005; Shepherd *et al*, 2005). Moreover, evidence indicates that the presence and severity of rash is correlated with survival in lung cancer (Perez-Soler *et al*, 2004), pancreatic cancer (Moore *et al*, 2005) and ovarian cancer (Gordon *et al*, 2005). As expected, AEs were more frequent with increasing doses of erlotinib in this trial. However, there was no evidence of cumulative toxicity as most patients completed the full six cycles of planned treatment.

Various clinical trials have also demonstrated that combining erlotinib with chemotherapy is feasible in a wide variety of tumours. In previously reported phase I/Ib trials, the MTD of erlotinib with cytotoxic agents ranged from 100 mg day^−1^ (with capecitabine/docetaxel: Trigo *et al*, 2003; with capecitabine/oxaliplatin: van Cutsem *et al*, 2003; with docetaxel: Mita *et al*, 2002) to 150 mg day^−1^ (with oxaliplatin/5-FU: Jones *et al*, 2003). The MTD of erlotinib of 75 mg day^−1^ in the present trial is lower than in trials with other combinations. This finding may be due to the additive toxicities with docetaxel/carboplatin. Indeed, dose escalation of erlotinib to 150 mg day^−1^ after chemotherapy suggests that erlotinib is better tolerated as a single agent in this patient setting.

Although preclinical and early clinical trials demonstrated promising antitumour activity of erlotinib in combination with chemotherapy, this benefit has not always been reflected in phase III trials. For example, first-line treatment with erlotinib (150 mg day^−1^) combined with standard platinum-based chemotherapy did not improve survival in the overall population of patients with advanced NSCLC; although never-smokers did experience a significant survival benefit in a post-randomisation analysis (Gatzemeier *et al*, 2004, 2005; Herbst *et al*, 2005). In contrast, erlotinib with gemcitabine prolonged survival somewhat in patients with previously untreated advanced pancreatic cancer compared with gemcitabine alone (Moore *et al*, 2005).

Erlotinib PK parameters were similar when erlotinib was given as a single agent or as part of the triple-drug combination, suggesting that there was no interaction between the agents, although the effect of erlotininb on the PK profiles of carboplatin and docetaxel was not directly assessed. However, in the current study, adding erlotinib to docetaxel/carboplatin resulted in an objective response rate of 52%; lower than that reported for docetaxel/carboplatin (59%) in our previously published phase III randomised trial (Vasey *et al*, 2004). In clinical trials evaluating first-line treatments for advanced ovarian carcinoma, the objective response rates (i.e. CR plus PR) have been 59% (docetaxel/carboplatin: Vasey *et al*, 2004), 59–68% (paclitaxel/carboplatin: Neijt *et al*, 2000; du Bois *et al*, 2003; Vasey *et al*, 2004) and 61–81% (paclitaxel/cisplatin: Neijt *et al*, 2000; du Bois *et al*, 2003). Possible explanations for the relatively limited antitumour efficacy of the triple-drug combination in this setting include the fact that the patient population was unselected for EGFR expression (although tissue samples were collected prior to study start for evaluation). In addition, there were only a small number of patients (*n*=45) included this trial, and thus appropriate conclusions regarding the efficacy of this regimen cannot be drawn.

In conclusion, 75 mg day^−1^ erlotinib was identified as the MTD in combination with standard doses of docetaxel (75 mg m^−2^) and carboplatin (AUC 5) on day 1 of 3-week cycles in patients with advanced, previously untreated ovarian cancer. Significant DLTs include rash and diarrhoea, which limit the usefulness of this combination regimen. Maintenance erlotinib following six cycles of combination chemotherapy is also associated with cutaneous toxicity, which can limit the dose and duration of treatment, although monotherapy does permit a higher dose to be given.

The potential benefit of this maintenance approach can only be addressed in a randomised trial. Under the auspices of the European Organisation for Research and Treatment of Cancer (EORTC), this is now being examined in a trial in which responding patients are randomised to erlotinib or control after completing platinum-based induction treatment. A parallel translational component to this international study will evaluate the possibility that patients most likely to derive benefit from erlotinib can be predicted by molecular tumour analysis. This phenomenon, in a similar manner to that seen in NSCLC, has been observed in a blinded molecular analysis of a Gynecologic Oncology Group (GOG) phase II trial in ovarian cancer with another EGFR inhibitor, gefitinib (Schilder *et al*, 2005). Results of the EORTC erlotinib trial are expected in 2009.

## Figures and Tables

**Table 1:** Baseline patient and disease characteristics

**Characteristics**	**Cohort 1**	**Cohort 2a**	**Cohort 2b**
Patients (*n*)	13	13	19
Median age (range) (years)	59.5 (47.9–68.3)	56.0 (32.4–65.7)	61.9 (49.1–68.7)
			
*ECOG PS (n)*			
0	6	5	4
1	7	8	15
			
*FIGO stage (n)*			
III	1	0	0
IIIa	0	1	0
IIIb	3	2	1
IIIc	6	7	15
IV	3	3	3
			
*Histology (n)*			
Serous adenocarcinoma	11	10	17
Endometroid carcinoma	1	0	0
Adenocarcinoma	1	2	2
Other^a^	0	1	0
			
*Disease bulk (n)*			
None or microscopic	3	2	7
<2 cm	6	4	2
⩾2 cm	4	5	6
Not available	0	2	4
RECIST evaluable disease at baseline	6	6	11

a Primary epithelial carcinoma of the ovary with hepatoid differentiation.

**Table 2:** Most common non-haematologic AEs (grades 3 and 4) occurring in more than one patient in any cohort, during treatment with chemotherapy

	**Cohort 1 (*n*=13)**		**Cohort 2a (*n*=13)**		**Cohort 2b (*n*=19)**	
**AE**	**Grade 3 *n* (%)**	**Grade 4 *n* (%)**	**Grade 3 *n* (%)**	**Grade 4 *n* (%)**	**Grade 3 *n* (%)**	**Grade 4 *n* (%)**
Diarrhoea	0	0	4 (31)	0	3 (16)	0
Fatigue	0	0	4 (31)	0	3 (16)	0
Rash/desquamation	0	0	3 (23)	0	2 (10)	0
Dehydration	0	0	1 (8)	0	3 (16)	0
Dysphagia	0	0	0	1 (8)	2 (10)	0
Nausea	0	0	3 (23)	0	0	0
Abdominal pain	0	0	1 (8)	0	1 (5)	0
Dermatology/skin other	1 (8)	0	0	0	1 (5)	0
Dizziness	0	0	1 (8)	0	1 (5)	0
Stomatitis/pharyngitis	0	0	0	0	2 (10)	0
Fainting	0	0	1 (8)	0	1 (5)	0
Vomiting	0	0	2 (15)	0	0	0

**Table 3:** Summary of grade 3 and 4 haematologic toxicities, during treatment with chemotherapy

	**Cohort 1 (*n*=13)**		**Cohort 2a (*n*=13)**		**Cohort 2b (*n*=19)**	
**Parameter**	**Grade 3 *n* (%)**	**Grade 4 *n* (%)**	**Grade 3 *n* (%)**	**Grade 4 *n* (%)**	**Grade 3 *n* (%)**	**Grade 4 *n* (%)**
Leucopaenia	9 (69)	0	4 (31)	6 (46)	13 (68)	3 (16)
Thrombocytopaenia	1 (8)	0	2 (15)	3 (23)	0	0
Anaemia	0	0	1 (8)	0	0	0
RBC transfusions	3	6	6			
Neutropaenia	2 (15)	9 (69)	1 (8)	12 (92)	2 (11)	16 (84)
Febrile neutropaenia	0	1 (8)	2 (15)	2 (15)	3 (16)	2 (11)
Infection/febrile neutropaenia	0	1 (8)	0	1 (8)	0	1 (5)

RBC transfusion threshold not mandated in protocol.

**Table 4:** PK parameters of erlotinib given alone or in combination with docetaxel and carboplatin (cohort 1)

**Parameter**	**Erlotinib alone (day −1)**	**Erlotinib in combination with docetaxel/carboplatin (day 1)**	**Erlotinib alone (day 7)**
*C*_max_ (ng ml^–1^)	922 (760–1119)	752 (624–906)	953 (738–1230)
*T*_max_ (h)	2.4 (1.5–3.9)	3.8 (1.9–7.6)	2.9 (2.0–3.6)
AUC_(0–24 h)_ (ng h ml^−1^)	10 520 (7482–14 791)	11 041 (8241–14 825)	11 246 (7762–16 293)

Data shown are geometric means (95% CIs) derived from the repeated measures analysis of variance, based on *n*=10 patients with full profiles on each day (other three patients did not have full profiles on each day).
